# iGTP: A software package for large-scale gene tree parsimony analysis

**DOI:** 10.1186/1471-2105-11-574

**Published:** 2010-11-23

**Authors:** Ruchi Chaudhary, Mukul S Bansal, André Wehe, David Fernández-Baca, Oliver Eulenstein

**Affiliations:** 1 Department of Computer Science, Iowa State University, Ames, IA 50011, USA; 2 The Blavatnik School of Computer Science, Tel Aviv University, Tel Aviv 69978, Israel; 3 Department of Electrical and Computer Engineering, Iowa State University, Ames, IA 50011, USA

## Abstract

**Background:**

The ever-increasing wealth of genomic sequence information provides an unprecedented opportunity for large-scale phylogenetic analysis. However, species phylogeny inference is obfuscated by incongruence among gene trees due to evolutionary events such as gene duplication and loss, incomplete lineage sorting (deep coalescence), and horizontal gene transfer. Gene tree parsimony (GTP) addresses this issue by seeking a species tree that requires the minimum number of evolutionary events to reconcile a given set of incongruent gene trees. Despite its promise, the use of gene tree parsimony has been limited by the fact that existing software is either not fast enough to tackle large data sets or is restricted in the range of evolutionary events it can handle.

**Results:**

We introduce iGTP, a platform-independent software program that implements state-of-the-art algorithms that greatly speed up species tree inference under the duplication, duplication-loss, and deep coalescence reconciliation costs. iGTP significantly extends and improves the functionality and performance of existing gene tree parsimony software and offers advanced features such as building effective initial trees using stepwise leaf addition and the ability to have unrooted gene trees in the input. Moreover, iGTP provides a user-friendly graphical interface with integrated tree visualization software to facilitate analysis of the results.

**Conclusions:**

iGTP enables, for the first time, gene tree parsimony analyses of thousands of genes from hundreds of taxa using the duplication, duplication-loss, and deep coalescence reconciliation costs, all from within a convenient graphical user interface.

## Background

The need to build species trees based on evidence from genes along entire genomes often arises in phylogenomic studies [1,2]. The problem is sometimes approached using supertree methods [3-5], which provide a way to combine several conflicting phylogenies on partially overlapping sets of taxa into a single comprehensive phylogeny. However, supertree methods (for example, the majority of those described in [6]) are typically designed to work with species trees, not gene trees, as their inputs. Unlike species trees, gene trees can contain more than one homolog of a gene from the same species. More crucially, genes are affected by complex evolutionary phenomena, such as deep coalescence (incomplete lineage sorting), gene duplication and subsequent loss, lateral gene transfer, and recombination, that can create tremendous heterogeneity in the topology of gene trees and obscure species relationships. One well-studied approach for dealing with these complications is *gene tree parsimony *(GTP) [7-18], which seeks a species tree that contains all taxa represented in the gene trees and implies the minimum reconciliation cost; that is, the fewest number of evolutionary events that explains the discordance among the gene phylogenies. We note that the term GTP has traditionally been used in the context of gene duplication and loss, but here we use it more generally to mean a method that tries to minimize some reconciliation cost. It should be mentioned that, in addition to GTP, there has also been considerable recent interest in probabilistic models of reconciliation [19-24]. Although these methods are beyond the scope of this paper, we point out that the main purpose of such techniques is typically not to produce species trees, but to construct gene trees or to identify discordance among gene trees.

**Figure 1:**
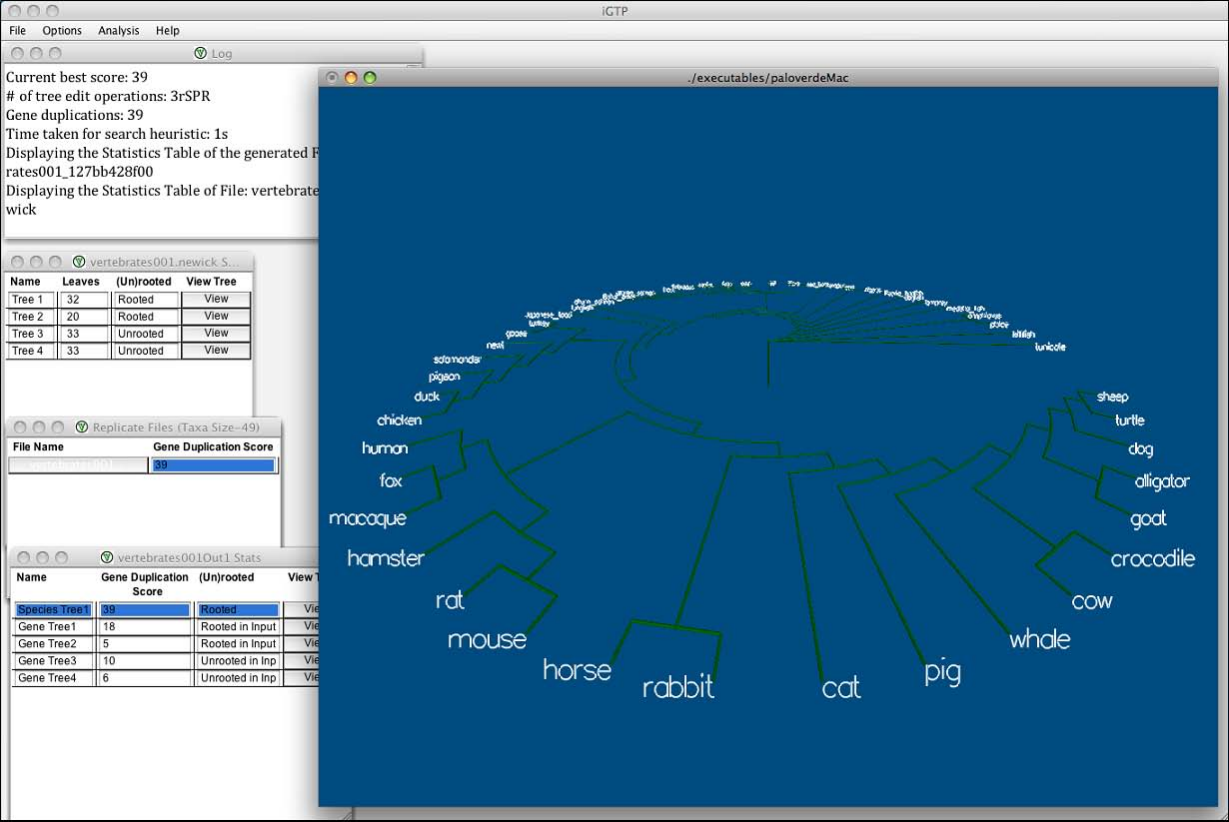
**main.png - Tree search in iGTP 1.1**. A sample execution of iGTP showing the input gene tree, output folder, and output file statistics windows. Also shown is a Paloverde window displaying a generated species tree.

While previous work suggests that GTP can produce accurate species trees [8,12-18], currently available software is either too slow to handle large data sets or lacks the flexibility to handle the wide range of evolutionary processes that affect gene tree topologies. Here we introduce iGTP, a stand-alone software application with an easy-to-use graphical user interface (Figure 1) that makes it possible to conduct large-scale gene tree parsimony analyses on hundreds of taxa and thousands of gene phylogenies for three of the most important variants of the GTP problem: (i) the duplication problem [7,25-32], which minimizes the number of gene duplications, (ii) the duplication-loss problem [7,25-34], which minimizes the number of gene duplications and losses, and (iii) the deep-coalescence problem [17,35,36], which minimizes the number of deep coalescences. All of these variants of GTP are intrinsically hard [37,38], and exact algorithms [15,17,39,40] are feasible only when there are very few taxa. Therefore, iGTP relies on widely-used local search heuristics that have been proven to be effective in previous studies [36,41,42]. iGTP simplifies the analysis of the results by displaying the reconciliation costs of the gene trees against the computed species trees in convenient tabular form, and by providing integrated options for displaying the gene trees and species trees.

iGTP's more advanced features include allowing unrooted gene trees in the input, assigning weights to the input gene trees, building effective initial trees using stepwise leaf addition, executing a constrained tree search by enforcing the presence of certain clades in the species tree, and automatically executing several replicates of the heuristic search. iGTP provides a scoring option that allows users to determine the total reconciliation cost of a given species tree with respect to a collection of gene trees.

Prior to iGTP, the three main software programs available for gene tree parsimony were Gene Tree [41], Mesquite [42], and DupTree [43]. iGTP deals with a broader range of evolutionary events than either Mesquite, which handles only deep coalescence, or DupTree, which handles only gene duplications. Further, neither GeneTree nor Mesquite can handle unrooted gene trees, but iGTP can. iGTP's scoring function is similar to one provided by the software program Notung [32,44]. While Notung performs scoring under the duplication-loss model, iGTP also handles pure duplication and deep coalescence. Also, Notung does not provide the option to compute supertrees. Further, iGTP can score multiple gene trees simultaneously against a species tree. On the other hand, Notung allows non-binary species trees or non-binary gene trees, but not both, while iGTP requires all trees to be binary.

iGTP combines DupTree with two new programs, DupLoss - for duplication and loss - and DeepC - for deep coalescence - under a common GUI. The use of these programs, which implement state-of-the-art algorithms, makes iGTP many times faster than GeneTree or Mesquite. Aside from the GUI, iGTP adds to DupTree the ability to perform replicate runs. While a preliminary version of DupLoss was mentioned in [11], that version could not handle unrooted or weighted gene trees, could not execute constrained or replicate searches, did not have options to build good initial species trees by stepwise taxon addition, and only implemented the most basic type of local search. Finally, this is the first time that the program DeepC has been implemented and made available.

The executable of iGTP, as well as its user manual, can be obtained at http://genome.cs.iastate.edu/CBL/iGTP/.

## Implementation

**Figure 2:**
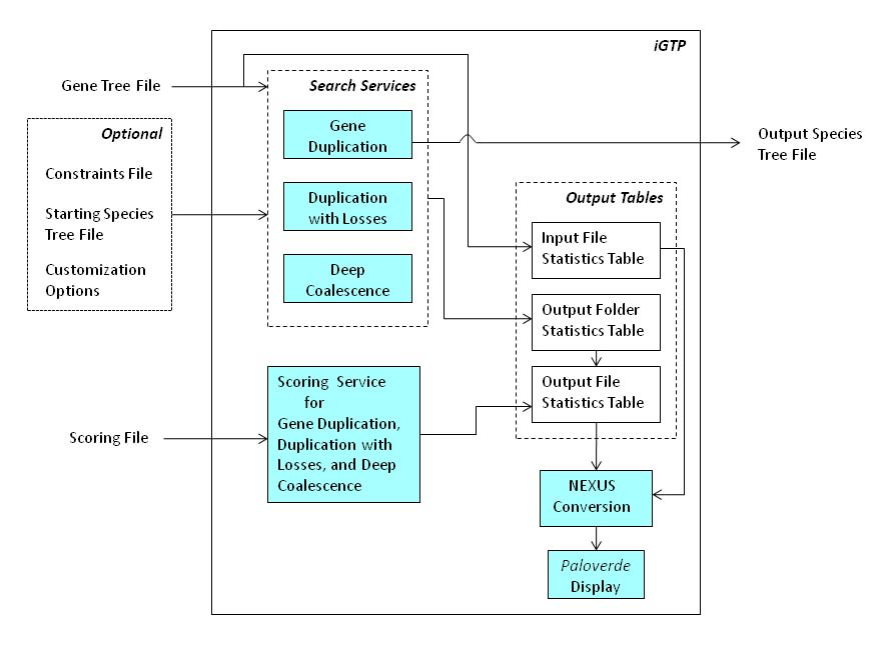
**arch1.png - Architecture Diagram of iGTP 1.1**. The rectangular box (labeled iGTP on the top-left corner) represents the user layer of iGTP. Inside this, the application layer services and output tables are shown by cyan and white boxes, respectively. The inputs are at the left and the outputs are at the right. The arrows indicate possible execution sequence.

Figure 2 illustrates the high-level system architecture of iGTP 1.1. The main design objective is flexibility. To accomplish this, the system is divided in a number of distinct modules, grouped into two layers: the user layer and the application layer. The former is implemented in Java Swing, which makes iGTP platform-independent. The application layer contains services for performing two kinds of operations: (i) searching for optimal species trees under the duplication, duplication-loss, and deep coalescence cost models, and (ii) scoring gene trees against a given species tree. All of these services are written in C++ for speed. Trees are displayed using the 3-D tree visualization tool Paloverde [45] after converting them into NEXUS format. Paloverde was chosen because of the ease with which it could be integrated into our system.

The input to a search operation is a gene tree file, which contains rooted or unrooted binary gene trees in Newick format. The input files are stored in the *inputData *folder under the project home directory. After the input is read, the user layer invokes the appropriate search service. The output is stored as a Newick file that contains all the optimal species trees, followed by all the input gene trees, as well as the total reconciliation cost of each species tree and the reconciliation costs of the individual gene trees against the species trees. This file can be found inside the *outputData *folder under the project home directory. The input to a scoring operation is a scoring file, which is comprised of a rooted species tree and a collection of rooted gene trees. All trees must be binary and in Newick format. The output of the scoring service is displayed on the screen.

## Results and Discussion

iGTP has an intuitive user interface that permits even novice users to immediately start gene tree parsimony analyses (see Figure 1). The interface items are divided into four categories. The *File *menu item allows opening and closing of input files. The *Analysis *menu item provides searching and scoring options. The user can customize the tree search by setting available options under the *Options *menu item. The *Help *menu item assists users with options available in iGTP. Moreover, tooltips are provided extensively to describe menu items over which the cursor is hovering.

**Figure 3:**
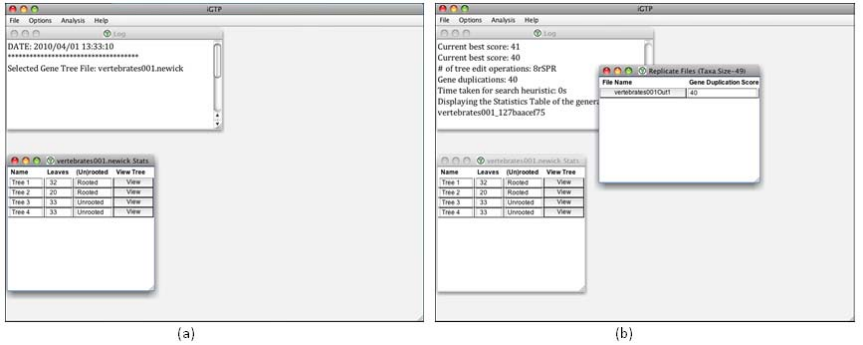
**execution.png - Sample execution of iGTP 1.1**. (a) iGTP displaying the statistics table for the selected input gene tree file. (b) After the tree search finishes, an output folder statistics table appears.

A basic (customization-free) tree search in iGTP consists of two steps: opening a gene tree file and triggering the appropriate search option (duplication, duplication-loss, or deep coalescence). The *Input File Statistics Table*, generated in the first step, lists each tree in the input gene tree file with its leaf count and rooting status; the tree itself can be displayed by clicking its *View *button. A successful tree search renders the *Output Folder Statistics Table*, listing the names of the generated output files and the scores of its species trees. Note that, in a basic execution of tree search, only one output file is generated. However, as described under "Customization Options", iGTP can run multiple replicates, leading to multiple output files. Each such file can be examined by clicking on its name. This displays the file's *Output File Statistics Table*, which lists the generated species tree followed by the input gene trees with their scores and rooting statuses. Trees can be displayed by clicking their *View *buttons (see Figure 1 and 3). A *Log *window displays the selections made in the drop-down menu options, and updates the user with intermediate results of the GTP search in real time.

A scoring operation consists of opening a scoring file and then selecting the appropriate scoring option (duplication, duplication-loss, or deep coalescence). The results of a scoring operation are displayed in a table that gives the total reconciliation cost for the input species tree, as well as the reconciliation cost between this tree and each of the input gene trees. As for tree search, any of the trees can be displayed by clicking its *View *button.

### Customization options

Under the *Options *menu item, iGTP offers various options for customizing tree search. The *Starting Species Tree Generation *item gives users three options to customize the initial species tree used for tree search. The *Leaf Adding *option utilizes a greedy stepwise addition algorithm. The *Random Tree *option starts the search with a random tree topology. The *User Specified *option allows users to supply their own starting trees.

The default name for the output folder is composed of a system generated unique hexadecimal number followed by the input gene tree file name. The user can choose a different name through the *Output Folder Name *submenu.

The *No. of Replicates *option allows the automatic execution of multiple heuristic searches on the same data set. This is done by pipelining the selected number of commands to the GTP search services. Each replicate uses a different random seed, allowing for a more thorough exploration of the search space. The *Constraints File *submenu allows one to impose constraints on the topology of the inferred species tree. To adjust the thoroughness of the tree search, iGTP provides three versions of the subtree pruning and regrafting (SPR) local search heuristic, which differ in the way they deal with multiple equally optimal trees. *Randomized Hill Climbing *randomly chooses one tree from among the optimal species trees in the SPR neighborhood and continues the local search step with it. The search terminates when none of the trees in the current local search step has a lower reconciliation cost. In contrast, in the *Partial Queue Based *approach, each optimal tree from the current local search step is enqueued and serves as the initial starting tree for the heuristic search until a better tree is found. Thus, a partial queue based heuristic terminates only if none of the local search steps, starting from each of the the enqueued trees, yields a tree with lower reconciliation cost. A more thorough version of the *Partial Queue Based *option is the *Queue Based *option, which enqueues all the optimal trees found so far, even if they were found in previous local search steps. As with the partial queue based heuristic, each of the latter trees serves as the initial starting tree for the heuristic search until a better tree is found. To adjust the behavior of this option, two more parameters, *Maximum Queue Size *and *No. of Trees*, are provided. The first parameter specifies the maximum number of trees that can be in the queue at any given time; the second sets the number of optimal trees to be output at the end.

The GTP method requires input gene trees to be rooted. Since it can often be hard to root gene trees accurately, the *Gene Tree Rooting *menu item provides two option for dealing with unrooted gene trees. The goal is to identify gene tree rootings that minimize the reconciliation cost. The *All *option examines the reconciliation cost of every possible rooting of each unrooted gene tree against each species tree encountered during the search. The *Optimal *option examines the reconciliation cost of every possible rooting of each unrooted gene tree only after the search reaches a local optimum. If rerooting the unrooted gene trees can reduce the reconciliation cost, then all unrooted gene trees are optimally re-rooted and the SPR heuristic search is repeated using the new rootings.

The setting for the random number generator seed used in the heuristics can be adjusted via the *Random Seed *option. This allows the user to select between a system generated number or a user-supplied number, which enables one to repeat a particular GTP search.

### Performance evaluation

To evaluate the performance of iGTP, we compared its running time to that of the program GeneTree [41], which implements SPR-based search heuristics for all three reconciliation costs considered in this paper. Since Mesquite [42] only allows GTP analyses under the deep coalescence reconciliation cost, we did not consider it in our study.

We created five different gene tree data sets with 50, 100, 200, 400, and 1000 taxa respectively. Each data set consisted of 20 gene trees with the same set of taxa and with random binary topologies and random assignment of leaf labels. All analyses were performed on a 3 Ghz Intel Pentium 4 CPU based PC with Windows XP operating system. Both iGTP and GeneTree were run using the randomized hill-climbing heuristic starting from the same user-given species tree.

Table 1 shows that iGTP outperforms GeneTree by a wide margin in terms of both running time and scalability. For instance, on the 200 taxon data set, iGTP was more than one thousand times faster for the duplication-loss and deep coalescence problems. Beyond 200 taxa, it became unfeasible to use GeneTree. Reference [18] demonstrates the utility of iGTP in performing accurate large-scale phylogenetic analyses. Specifically, it uses the algorithms and features implemented in iGTP to perform a genome-scale phylogenetic analysis of 136 plant taxa using 18,896 nuclear gene trees.

**Table 1:** iGTP and GeneTree. Comparison of the run-times of iGTP and GeneTree on the same randomly generated data sets. Times are given in days(d), hours(h), minutes(m), and seconds(s).

Taxa	Duplication		Duplication-Loss		Deep Coalescence	
	iGTP	GeneTree	iGTP	GeneTree	iGTP	GeneTree
50	3 s	9 m:23 s	11 s	11 m:42 s	8 s	11 m:18 s
100	13 s	3 h:25 m	42 s	3 h:57 m	26 s	3 h:16 m
200	1 m:47 s	4 d:12 h:33 m	5 m:00 s	5 d:19 h:49 m	3 m:39 s	3 d:16 h:42 m
400	13 m:28 s	-	39 m:57 s	-	26 m:01 s	-
1,000	3 h:47 m	-	20 h:16 m	-	18 h:57 m	-

## Conclusion

Genome-scale phylogenetic analyses must account for complex evolutionary processes such as gene duplication and loss, incomplete lineage sorting (deep coalescence), or horizontal gene transfer, that can create incongruence among gene trees. iGTP is a software tool that enables, for the first time, rigorous, large-scale gene tree parsimony analyses based on thousands of genes using the duplication, duplication-loss, and deep coalescence reconciliation costs, all from within a convenient and user-friendly graphical interface.

## Availability and requirements

**Project name: **iGTP

Project home page: http://genome.cs.iastate.edu/CBL/iGTP/

**Operating system(s): **Platform independent, and tested on Linux, Mac OS X (10.4.11, 10.5.8), Microsoft Windows (XP, Vista, and 7).

**Programming languages: **Java Swing and C++

**Other requirements: **Java Runtime Environment version 5 or higher, at least 512 MB of main memory (recommended), and a modern 3D capable graphics card for visualizing large trees.

**License: **None

**Any restrictions to use by non-academics: **None

## Authors' contributions

RC and MSB contributed equally to this work. RC developed the graphical user interface and integrated it with the back-end C++ programs, wrote the project webpage, and contributed to the writing of the manuscript and the manual. MSB implemented the C++ programs for performing GTP analyses using the duplication-loss and deep coalescence costs, and contributed to the writing of the manuscript and the manual. AW helped with the development of the GUI, helped integrate Paloverde with iGTP, and wrote the back-end tools for scoring a given species tree. OE and DFB supervised the project and contributed to the writing of the manuscript. All authors read and approved the final manuscript.
